# The spectrum of *KIAA0196* variants, and characterization of a murine knockout: implications for the mutational mechanism in hereditary spastic paraplegia type SPG8

**DOI:** 10.1186/s13023-015-0359-x

**Published:** 2015-11-16

**Authors:** Amir Jahic, Mukhran Khundadze, Nadine Jaenisch, Rebecca Schüle, Sven Klimpe, Stephan Klebe, Christiane Frahm, Jan Kassubek, Giovanni Stevanin, Ludger Schöls, Alexis Brice, Christian A. Hübner, Christian Beetz

**Affiliations:** Department of Clinical Chemistry and Laboratory Medicine, Jena University Hospital, Jena, Germany; Institute of Human Genetics, Jena University Hospital, Jena, Germany; Hans Berger Department of Neurology, Jena University Hospital, Jena, Germany; Hertie-Institute for Clinical Brain Research, Department of Neurodegenerative Diseases, University of Tübingen, Tübingen, Germany; German Research Center for Neurodegenerative Diseases (DZNE), Tübingen, Germany; Dr. John T. Macdonald Foundation Department of Human Genetics and John P. Hussman Institute for Human Genomics, Miami, FL USA; Department of Neurology, University Medical Center of the Johannes-Gutenberg University Mainz, Mainz, Germany; Department of Neurology, University Hospital, Freiburg, Germany; Department of Neurology, University of Ulm, Ulm, Germany; INSERM U1127, Sorbonne Universités, UPMC Univ Paris 06 UMR_S1127, CNRS UMR7225, EPHE, Institut du Cerveau et de la Moelle épinière, Paris, France

**Keywords:** Hereditary spastic paraplegia, KIAA0196, Mouse model, Knockout, SPG8, Strumpellin

## Abstract

**Background:**

The hereditary spastic paraplegias (HSPs) are rare neurodegenerative gait disorders which are genetically highly heterogeneous. For each single form, eventual consideration of therapeutic strategies requires an understanding of the mechanism by which mutations confer pathogenicity. SPG8 is a dominantly inherited HSP, and associated with rather early onset and rapid progression. A total of nine mutations in *KIAA0196*, which encodes the WASH regulatory complex (SHRC) member strumpellin, have been reported in SPG8 patients so far. Based on biochemical and cell biological approaches, they have been suggested to act via loss of function-mediated haploinsufficiency.

**Methods:**

We generated a deletion-based knockout allele for *E430025E21Rik,* i.e. the murine homologue of *KIAA0196*. The consequences on mRNA and protein levels were analyzed by qPCR and Western-blotting, respectively. Motor performance was evaluated by the foot-base angle paradigm. Axon outgrowth and relevant organelle compartments were investigated in primary neuron cultures and primary fibroblast cultures, respectively. A homemade multiplex ligation-dependent probe amplification assay enabling identification of large inactivating *KIAA0196* deletion alleles was applied to DNA from 240 HSP index patients.

**Results:**

Homozygous but not heterozygous mice showed early embryonic lethality. No transcripts from the knockout allele were detected, and the previously suggested compensation by the wild-type allele upon heterozygosity was disproven. mRNA expression of genes encoding other SHRC members was unaltered, while there was evidence for reduced SHRC abundance at protein level. We did, however, neither observe HSP-related *in vivo* and *ex vivo* phenotypes, nor alterations affecting endosomal, lysosomal, or autophagic compartments. *KIAA0196* copy number screening excluded large inactivating deletion mutations in HSP patients. The consequences of monoallelic *KIAA0196*/*E430025E21Rik* activation thus differ from those observed for dominant HSP genes for which a loss-of-function mechanism is well established.

**Conclusions:**

Our data do not support the current view that heterozygous loss of strumpellin/SHRC function leads to haploinsufficiency and, in turn, to HSP. The lethality of homozygous knockout mice, i.e. the effect of complete loss of function, also argues against a dominant negative effect of mutant on wild-type strumpellin in patients. Toxic gain-of-function represents a potential alternative explanation. Confirmation of this therapeutically relevant hypothesis *in vivo*, however, will require availability of appropriate knockin models.

**Electronic supplementary material:**

The online version of this article (doi:10.1186/s13023-015-0359-x) contains supplementary material, which is available to authorized users.

## Background

Hereditary spastic paraplegia (HSP) refers to a group of rare disorders in which progressively spastic gait is the major and often primary symptom. The pathological correlate of this phenotype is a distal-to-proximal degeneration of upper motoneuron axons in the corticospinal tract [1]. While HSPs have long been categorized according to age at onset, mode of inheritance, and presence/absence of additional symptoms, a genetic classification scheme is currently taking over. To date, more than 70 spastic paraplegia gene loci (SPGs) have been reported [2, 3].

HSP type SPG8 is an autosomal dominant form which is associated with a comparatively young age at onset and a pure, but rather severe phenotype [4, 5]. In 2007, *KIAA0196* mutations were identified in six SPG8 pedigrees [5], but only six more families have been published since [6–10]. *KIAA0196* codes for strumpellin, a 1159-residue protein which contains a short central spectrin repeat but otherwise seems to lack recognizable homology domains [5]. Strumpellin is a member of the multiprotein WASH regulatory complex (SHRC) [11, 12]. This complex associates with retromer, another multi-protein complex, and regulates the tubular extension of early endosomes [11, 13–16]. It may thereby facilitate cargo sorting for endosome-to-Golgi retrieval, for membrane receptor recycling and/or for targeting to the lysosomal degradative pathway [11, 17, 18]. Distinct additional roles in autophagy have been proposed more recently [19–21].

Eight unique *KIAA0196* missense mutations have been associated with HSP so far [5–10]. By affecting residues 226, 471, 583, 591, 619, 620, 626, and 696, they seem to cluster in the protein’s central part. Interestingly, an overlapping central region is also affected by a genomic deletion of exons 11–15 (encoding residues 470–672) [8]. Functional assays have been performed for some of the missense variants, but did not reveal any alterations regarding subcellular localization, interaction potential, SHRC assembly, retromer binding, and endosomal tubulation [12, 22, 23]. In contrast, RNAi-mediated knockdown of strumpellin was found to have strong effects in cell lines [11, 14, 22, 23] and in zebrafish embryos including abnormal development of spinal cord motoneurons [5, 22]. Collectively, these findings have been interpreted in light of the mutational mechanism relevant for SPG8: they were suggested to argue against a dominant negative effect of mutant strumpellin alleles on the wild-type allele, but to, instead, indicate loss-of-function-mediated haploinsufficiency [8, 22, 23]. Against this background, the apparent absence of classical loss-of-function mutations (i.e. non-sense, frame-shift, splice-site, whole gene deletions) in SPG8 patients was attributed to a lack of appropriate tools (e.g. for detecting deleted alleles) and/or compensation by the non-inactivated allele [23].

In the present study we used genetic and *in vivo* approaches to further elucidate the potential mechanisms by which mutations in *KIAA0196* cause HSP. Our findings strongly question the current haploinsufficiency hypothesis. As they also provide additional evidence against relevance of a dominant negative effect of mutant on wild-type strumpellin, we discuss alternatives and provide a conceptual basis for experimental testing.

## Methods

### Generation of mouse lines

**Fig. 1:**
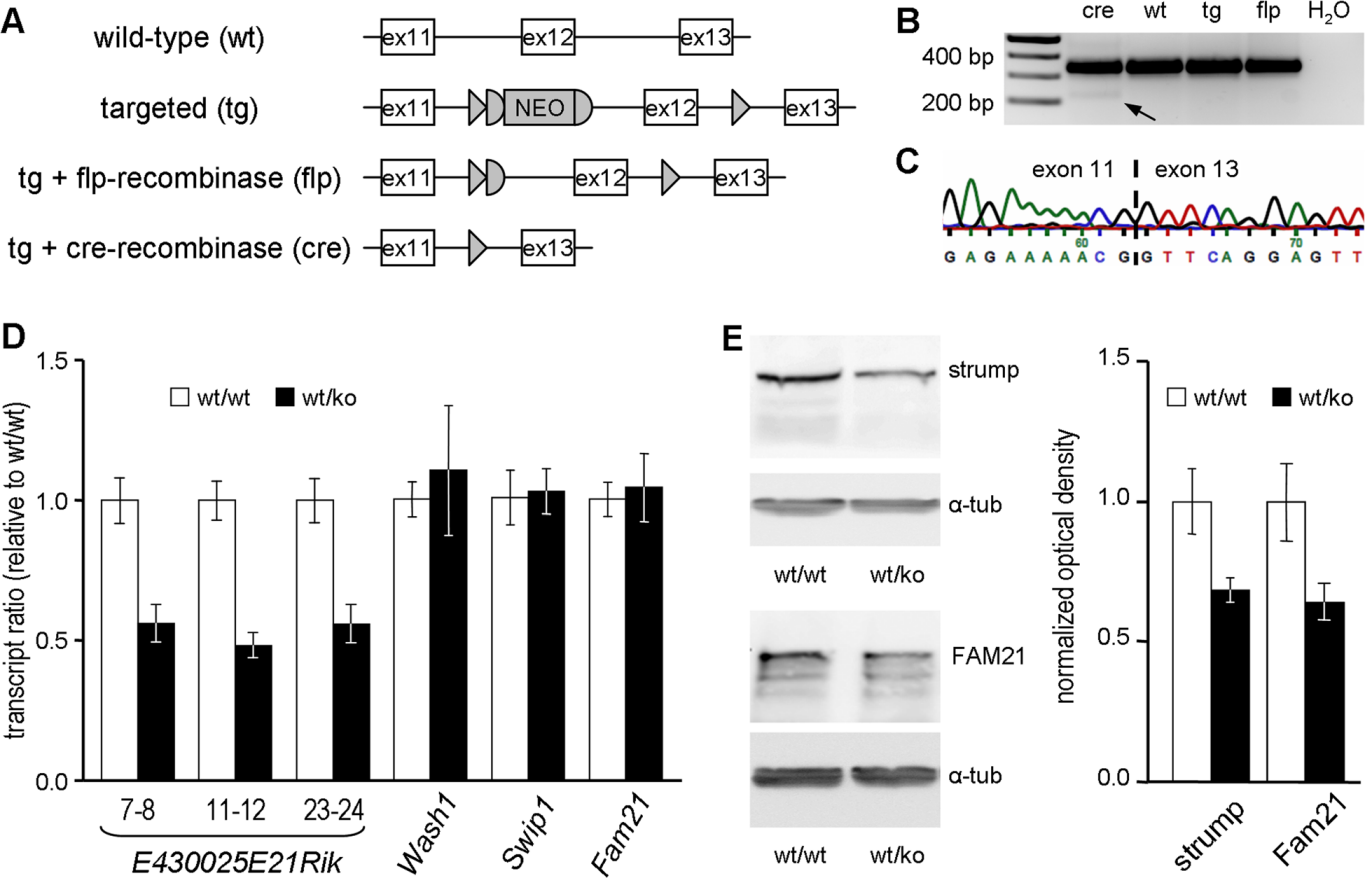
Targeted deletion of *E430025E21Rik* exon 12, and determination of consequences at mRNA and protein levels. **a** Wild-type (“wt”) and targeted allele (“tg”), and recombinase-mediated generation of the conditional (“flp”) and the constitutive (“cre”) deletion. ex, exon; NEO, neomycin resistance cassette; triangles, loxP sites; half-circles, frt sites. **b** RT-PCR on brain-derived cDNA with forward and reverse primers in exon 11 and exon 13, respectively. Note the smaller extra product (arrow) which is specific to cre-derived template. **c** Sequence analysis of product marked in (B). **d** qPCRs on brain-derived cDNA. For *E430025E21Rik*, the exons targeted in three distinct assays are indicated. Diagrams depict normalized geometrical means (n = 3); error bars represent SEM. **e** Western blot-based quantification of strumpellin and Fam21 in brain lysates after normalization to α-tubulin. Diagram depicts normalized means (n = 3), error bars represent SEM

By screening of a 129/SvJ mouse genomic λ library (Stratagene) with a probe derived from intron 14 of *E430025E21Rik*, i.e. the murine homologue of *KIAA0196*, we obtained a 7.6 kb *Spe*I fragment which spans introns 9 to 15. This fragment was cloned into the pKO-V901 plasmid (Lexicon Pharmaceuticals, The Woodlands, TX) which contains a phosphoglycerate kinase (pgk) promoter-driven diphtheria toxin A cassette as a negative selection marker. Applying several sub-cloning steps, a single loxP site was inserted into an *Nsp*I site 216 bp downstream of exon 12, while an *Asc*I site was placed 265 bp upstream of exon 12 and used for inserting a pgk promoter-driven neomycin resistance cassette flanked by frt-sites and preceded by another loxP site (Fig. 1a). Following electroporation of this targeting construct into embryonic stem (ES) cells according to standard procedures, Neomycin-resistant clones were analyzed by Southern Blotting of *Xba*I-digested ES cell DNA. One of the positive clones was injected into C57BL6 blastocysts. The resulting chimeras were subsequently backcrossed with C57BL6 to obtain heterozygously targeted mice. Mating with lines that express either cre- or flp-recombinase resulted in the heterozygous constitutive and conditional exon 12 deletion lines, respectively (Fig. 1a). Studies were performed with littermates of the F3 generation. Genotypes were determined by PCR on DNA isolated from tail biopsies (primer sequences available upon request). All animal experiments were approved by the responsible local authorities (TLV, internal reference “SPG8 Mäuse”).

### Quantitative PCR and western blotting

Mice were anesthetized and perfused with 25 ml PBS. Brains were removed and snap-frozen in liquid nitrogen. For quantitative PCR (qPCR), 30 mg of cerebral cortex were homogenized in a glass-glass potter and total RNA isolated with the RNeasy kit (Qiagen, Hilden, Germany) following the manufacturer’s instructions. qPCR was performed in a 20 μl amplification mixture consisting of Brilliant® II SYBR Green QPCR Master Mix (Stratagene, La Jolla, CA), cDNA (equivalent to 25 ng reverse-transcribed RNA) and primers (250 nM final concentration each; sequences available upon request). Transcripts were amplified with Rotor Gene 6000 (Corbett Life Science, now Qiagen). Expression levels were calculated using the Pfaffl equation [24], normalized against *Tubb3*, and geometrical means from three animals expressed as fractions of wild-type values. For Western blotting, brain hemispheres were homogenized in RIPA lysis buffer containing protease inhibitor cocktail cOmplete (Roche Diagnostics, Rotkreuz, Switzerland), and centrifuged at 13.200 rcf for 3 min. 10 μl of supernatant were loaded on 10 % polyacrylamide gels and transferred onto PROTRAN nitrocellulose membranes (Whatman GmbH, Dassel, Germany). Blots were probed with antibodies against strumpellin (C-14, Santa Cruz Biotechnology, Dallas, TX), FAM21 [25] (antiserum kindly provided by Matthew N. Seaman), and α-tubulin (T9026, Sigma-Aldrich, St. Louis, MO), and signals obtained by applying appropriate horseradisch peroxidase-conjugated secondary antibodies. Quantification of signal intensity was done by standard densitometry of scanned Western blot images.

### Phenotyping

**Fig. 2:**
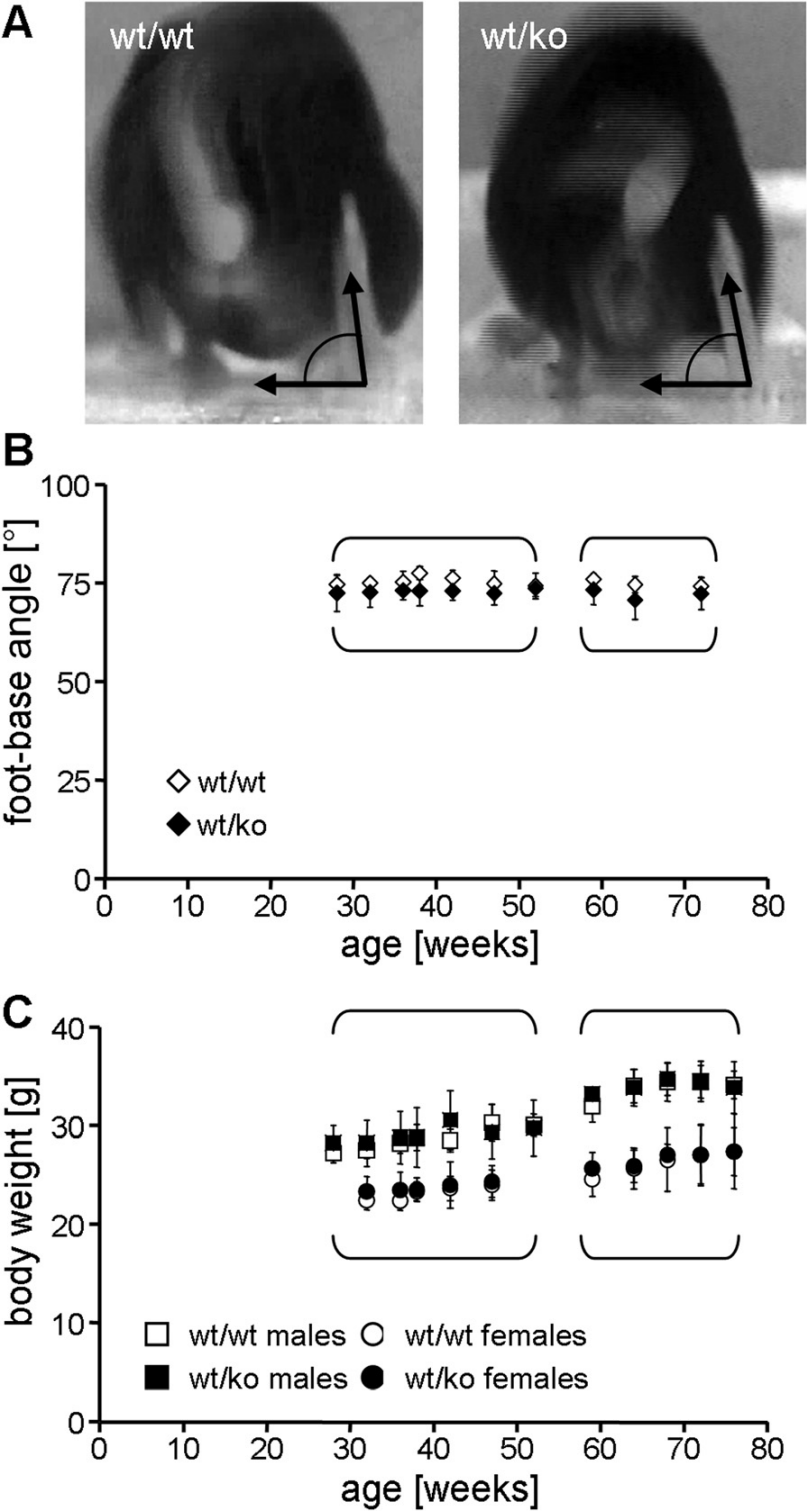
Comparative phenotyping of wild-type vs. heterozygous *E430025E21Rik* animals in two independent cohorts. **a** Determination of the foot-base angle (FBA) by videotaping the beam-traversing animal from behind. **b** FBA over time. Brackets denote cohort identity (n = 3-13 for younger cohort; n = 9 for older cohort); error bars represent SD. **c** Body weight for males and females. Animal identity, brackets and error bars as in (**b**)

Two independent cohorts of animals (aged 28–52 weeks and 59 to 76 weeks, respectively) were investigated every four to five weeks. The foot-base angle, previously shown to be highly sensitive in detecting HSP-related gait abnormalities in mice [26, 27], was applied as described [28]. Briefly, the angle which hind-paw and surface enclose at toe-off positions was quantified using single video frames from recordings of beam walking mice (Fig. 2a). 12 randomly chosen steps from two to three consecutive runs were considered. Prior to starting monthly phenotyping, animals were weighted. The significance of differences between genotype-specific values was analyzed timepoint-wise by the 2-sided Student’s *T*-test after Bonferroni correction.

### Primary cell cultures

Cortical neurons were prepared from P0 or P1 animals, and cultured as described [29]. Cells were fixed after 96 h and immunostained for the pan-axonal neurofilament marker SMI312. Images of neurons with SMI312 positive neurites (i.e. axons) were acquired at 40x magnification. Axonal branches were counted, and axon length was measured in ImageJ; means for genotypes were compared by the two-sided Student’s *T*-test.

Mouse adult fibroblasts (MAFs) were prepared from 2-months-old mice following a standard protocoll [30]. Cells were grown for 10 days, seeded into 8-well chamber slides (Lab-Tek™; ThermoFisher Scientific, Waltham, MA), and grown for another 2 days. For transfection of an LC3-RFP construct (kindly provided by C. Kaether) lipofectamine (Invitrogen, now Life Technologies, Carlsbad, CA) was used. Cells were fixed in 4 % paraformaldehyde, permeabilized in 0.1 % Triton X-100/PBS for 15 min, and incubated in blocking buffer containing goat serum for 30 min. Endosomes and lysosomes were visualized by immunfluorescence against EEA1 (Ab2900; Abcam, Cambridge, UK) and LAMP-1 (553792; BD Pharmigen, CA, USA), respectively. Anti Snx1 (sc-10609; Santa Cruz Biotechnology, Dallas, TX) was used to specifically visualize endosomes with protruding tubulations. Images were obtained by confocal laser scanning microscopy (LSM710 Meta; Zeiss, Oberkochen, Germany).

### Patients and controls

Two independent cohorts of HSP index patients were analyzed. The first one consisted of 148 patients of mainly French origin. Pure and complex HSP had been diagnosed in 74 cases each. Family history was consistent with dominant inheritance in 138, while 10 cases were sporadic. All patients were negative for mutations in *SPAST* (SPG4), while mutations in *ATL1* (SPG3A) had only been excluded when age at onset was <20 years. The second cohort consisted of 92 patients (42 and 50 with pure and complex HSP, respectively) from central European countries. In 83 cases there was evidence for dominant inheritance (two successive generations affected), while the remaining cases were compatible with both dominant and recessive inheritance. Informed consent had been obtained prior to genetic analysis from all patients. Anonymized DNA samples from unrelated projects were used as controls.

### KIAA0196-specific multiplex ligation-dependent probe amplification assay

Genomic sequence of the human *KIAA0196* gene was downloaded from the USCS genome browser (www.genome.ucsc.edu), and all 29 exons of the major isoform (NM_014846) marked. A total of six exonic MLPA probes were placed along the gene such as to achieve roughly equal spacing (Fig. 4a). Probe design was done according to criteria provided by MRC-Holland (The Netherlands) at www.mlpa.com (see Additional file 1: Figure S1 for sequences of the corresponding MLPA probe oligonucleotides). Six reference probes targeting physically distinct genomic regions were derived from previously established probesets ([31, 32], unpublished). Oligonucleotides for MLPA probes were from MWG Eurofins (Ebersberg, Germany). MLPA reactions utilised reagents from MRC-Holland, and products were visualised on a LICOR4200 (LICOR Biosciences, Lincoln, NE). Relative MLPA signals were calculated as described previously [33].

## Results

### Genomic deletion of KIAA0196 exon 12 creates a homozygously lethal knockout allele which reduces SHRC abundance in heterozygosity

In order to model loss of strumpellin function in mice, we decided for a classical cre/loxP strategy which targets exon 12 of the murine *KIAA0196* homologue *E430025E21Rik* (Fig. 1a). F1 animals carrying one exon 12-deleted allele were viable and fertile, and gave heterozygous offspring in the expected 50 % ratio when crossed back with C57BL/6 wild-type animals. Matings in which both parents were heterozygous (henceforth referred to as *E430025E21Rik*^wt/ko^ animals) failed to result in homozygous offspring. As this suggested prenatal lethality, we analyzed embryonic stages E19.5, E16.5, and E13.5. While *E430025E21Rik*^wt/wt^ and *E430025E21Rik*^wt/ko^ were generally found in a ~1:2 ratio, not a single *E430025E21Rik*^ko/ko^ was detected amongst 63 embryos (Table 1), and no signs of terminated embryonic development were observed in dissected uteri. This suggests that strumpellin is necessary for survival of very early embryonic stages and/or for attachment of blastocyts to the epithelial lining of the maternal uterus.

**Table 1:** Distribution of genotypes obtained from *E430025E21Rik* ^wt/ko^ x *E430025E21Rik* ^wt/ko^ matings

	Genotype			
Age	wt/wt	wt/ko	ko/ko	*p*-value
P1	12	25	0	2.4 x 10^−5^
E19.5	2	3	0	0.23
E16.5	3	8	0	0.042
E13.5	4	6	0	0.056
Total	21	42	0	1.3 x 10^−8^
Ratio	1	2	0	

In brain-derived cDNA of heterozygous animals, RT-PCR with primers in exon 11 and exon 13 resulted in two bands: one band of normal size and one additional smaller one (Fig. 1b). The latter contributed less than 0.1 % of total product, was absent in wild-type samples, and represented an mRNA species in which exon 13 directly follows exon 11 as confirmed by sequence analysis (Fig. 1c). The weakness of this RT-PCR signal is likely explained by non-sense mediated decay due to presence of a pre-terminal stop codon in the altered exon 13 reading frame. The exon 12 deletion thus created a true knockout allele.

Heterozygous inactivation of a gene may result in up-regulation of expression from the wild-type allele. We therefore designed a set of *E430025E21Rik*-specific qPCR primers, and analyzed brain-derived cDNA. Our finding of a ~50 % reduction in the total amount of *E430025E21Rik* transcript (Fig. 1d) in heterozygous animals indicates that there is no compensation by the wild-type allele at mRNA level. With enhanced protein stability representing another potential compensatory mechanism we also analyzed brain lysates by Western blotting, and found that the reduction in *E430025E21Rik* mRNA abundance directly translates into reduced abundance of strumpellin protein (Fig. 1e). A similar degree of reduction was observed for the SHRC core protein Fam21 (Fig. 1e), while mRNA expression of several genes encoding SHRC members was unaltered (Fig. 1d). Heterozygous presence of an *E430025E21Rik* knockout allele thus results in less strumpellin protein and a correspondingly reduced abundance of the multi-protein SHRC complex.

### A reduction in strumpellin/SHRC levels does not entail HSP-related phenotypes

In order to determine whether the observed reduction in the amount of strumpellin/SHRC results in a movement phenotype we performed foot-base angle measurements (Fig. 2a), i.e. a paradigm which has proven very sensitive in revealing early onset, progressive gait impairment in murine models for other forms of HSP (e.g. [26, 27]). The foot-base angles in heterozygous animals did not differ from those of wild-type siblings (Fig. 2b). Also in contrast to what is observed in many other HSP models (e.g. [27, 34]), body weight for both sexes was normal at all ages (Fig. 2c). Collectively, these data suggested that the reduced amount of strumpellin/SHRC is sufficient for maintaining a normal overall phenotype, particularly normal motor performance.

### Ex vivo axon outgrowth as well as endosomal tubulation and the integrity of organelle compartments are unaltered upon heterozygous presence of an E430025E21Rik knockout allele

**Fig. 3:**
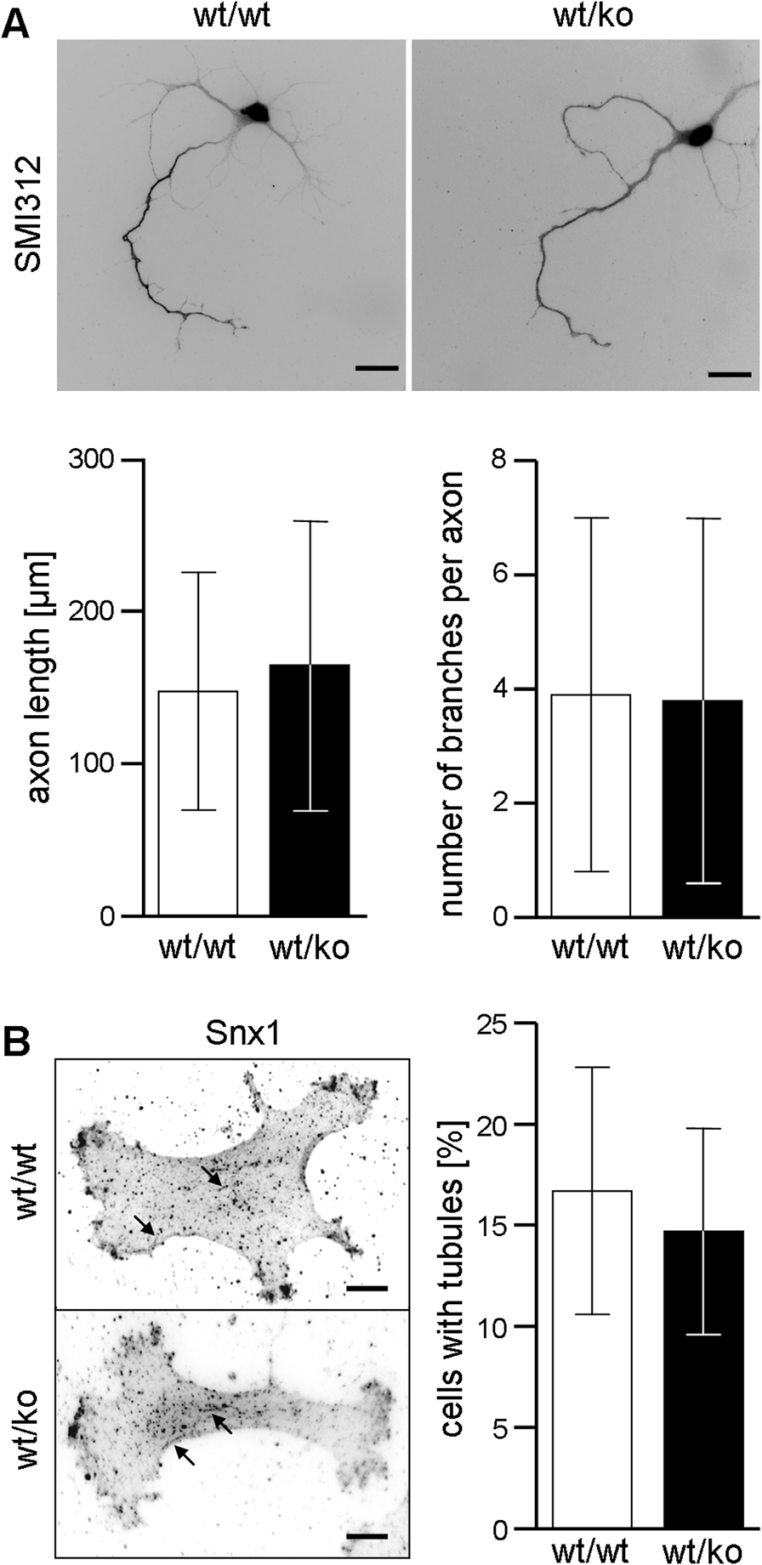
Investigation of potential consequences of heterozygous *E430025E21Rik* knockout *ex vivo*. **a** Quantification of axon length and axonal branching in primary cortical neurons which were stained for the axon marker SMI312 96 h after plating. Scale bars: 20 μm; error bars represent SD. **b** Visualization of endosomal tubules (arrows) in primary mouse adult fibroblasts by immunofluorescence against the retromer subunit Snx1. The fraction of cells with at least one tubule was determined in 30–40 cells in three independent experiments. Scale bars: 10 μm; error bars represent SD

Although HSP is considered a degenerative rather than a developmental disease [1], *ex vivo* axon outgrowth assays have revealed aberrations for numerous clinically valid mouse models [27, 35, 36]. We therefore cultured cortical neurons from P1 animals and stained them for an axon marker. After four days, i.e. when significant alterations are already obvious in neurons from other models (e.g. [27]), we did not detect any abnormalities including for critical parameters such as total axon length and number of axonal branches (Fig. 3a). Heterozygosity for an *E430025E21Rik* knockout allele does therefore not negatively impact on axon outgrowth.

Our protein level observations argued for a reduced amount of total SHRC in heterozygotes *in vivo* (see Fig. 1e). Completely ablating SHRC has been shown to result in increased endosomal tubulation [11, 23], in the collapsing of the endosolysosomal network [17], and in a strong de-regulation of autophagy [19–21]. We therefore cultured mouse adult fibroblasts (MAFs) from wild-type and from heterozygous strumpellin knockout mice, and investigated the corresponding organelle compartments. Immunfluorescence against Snx1 revealed similar fractions of cells containing tubulated endosomes (Fig. 3b). Based on the general endosomal and lysosomal markers EEA1 and LAMP1, respectively, these compartments appeared completely normal as regards organelle size, number, and cellular distribution (Additional file 2: Figure S2). Similarly, transfection of a fluorescently tagged fusion protein of the autophagosome/autolysosome marker LC3 failed to reveal any autophagy-related abnormalities (Additional file 2: Figure S2). Our moderate reduction of strumpellin/SHRC levels, thus, does not trigger the drastic morphological abnormalities observed upon complete ablation*.*

### Genetics further questions the strumpellin/SHRC haploinsufficiency hypothesis for HSP

**Fig. 4:**
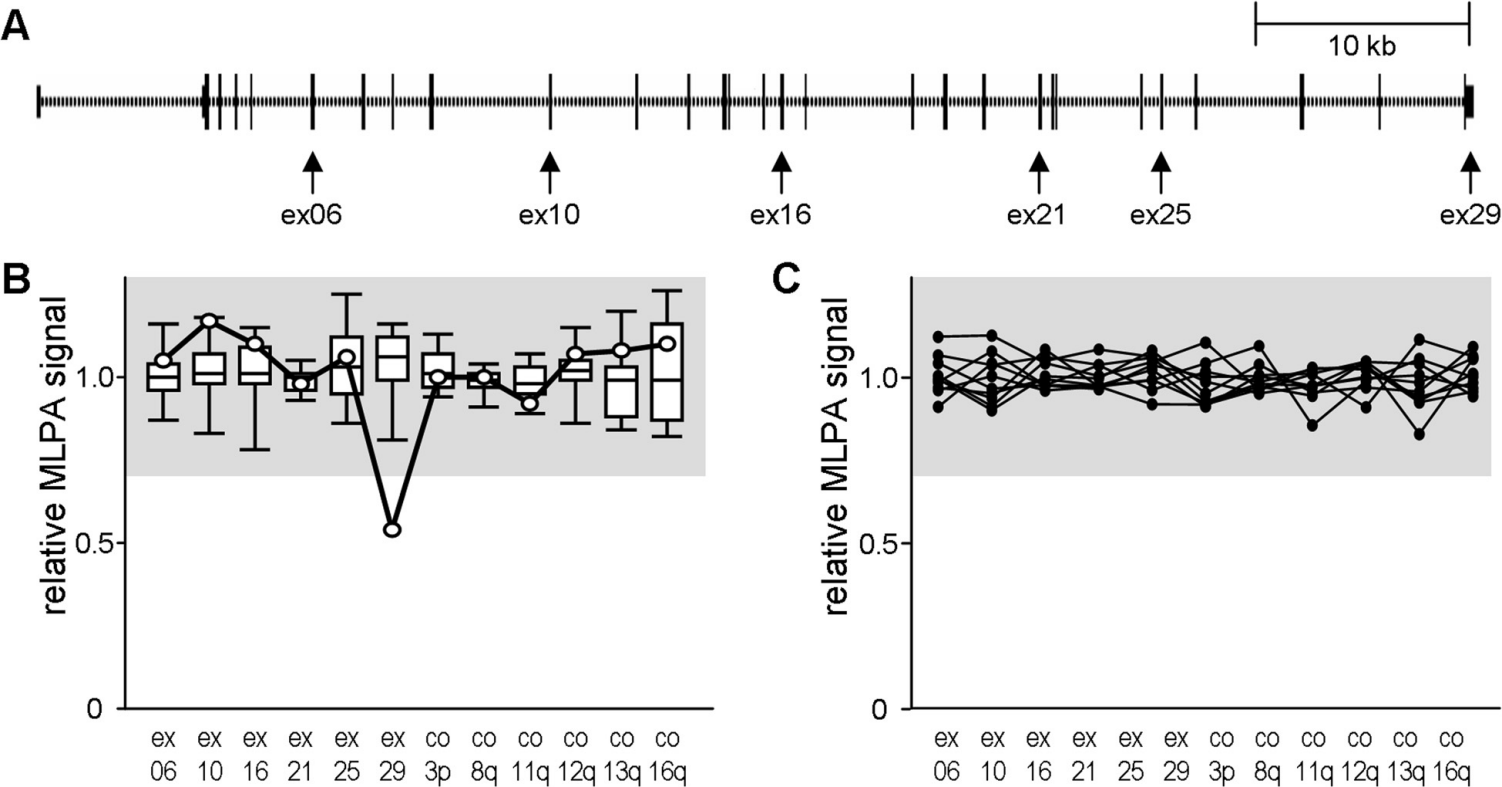
*KIAA0196* copy number screening by multiplex ligation-dependent probe amplification (MLPA). **a** To scale scheme of *KIAA0196* gene with exons represented by vertical bars. Arrows indicate targets of MLPA probes. ex, exon. **b** validation of MLPA probe mix on 12 control DNAs (summarized as box plots) and on a sample known to carry a rare SNP at the ligation site for the exon 29-specific probe (circles). The 0.7 to 1.3 signal range indicative of normal copy number is marked in grey. co, control MLPA probes targeting independent chromosomal arms as indicated. **c** Exemplary results for ten HSP index patients

In parallel to generating a murine *E430025E21Rik* knockout allele, we aimed at screening HSP patients for bona fide knockout mutations. We reasoned that deletions encompassing the whole gene or at least its 5′ part would represent such definite loss-of-function alleles. As these genomic rearrangements are not detectable by standard sequencing-based approaches, we developed a *KIAA0196*-specific MLPA kit (Fig. 4a). We first validated it on a set of control DNAs and on a sample carrying a rare SNP at one of the MLPA probes’ ligation site (Fig. 4b). The application to 240 samples from HSP index patients, however, did not identify any *KIAA0196* copy number aberration (Fig. 4c).

Considering that neither small truncating mutations nor large inactivating deletions are apparently present in HSP patients we aimed at a comparison with other dominant HSP genes that are associated with clinically pure HSP. For *SPAST* (SPG4) and *REEP1* (SPG31), i.e. two frequently involved genes [37, 38], haploinsufficiency is well-supported [26, 39]. In contrast to *KIAA0196*, stop mutations, small indels, and large inactivating deletions of either *SPAST* or *REEP1* are frequently found in HSP patients (Table 2). Also of note is the absence of small *SPAST* and *REEP1* truncating variants in a large cohort of individuals presumably not suffering from HSP, compared to the presence of three such *KIAA0196* variants (Table 2). These differences regarding the spectrum of mutations and variants further argue for a difference regarding the mutational mechanism.

**Table 2:** Mutation and variation spectra for selected dominant HSP genes

Gene (HSP sybtype)	*SPAST* (SPG4)	*REEP1* (SPG31)	*KIAA0196* (SPG8)
Relevant pathomechanism	Haploinsufficiency	Haploinsufficiency	(controversial)
Large inactivating deletion mutations in HSP patients	yes [33]	yes [44]	no [present study]
Fraction of small stop/indel mutations in HSP patients	~45 % [45]	~60 % [44, 46]	0 % [10, and references therein]
Small stop/indel variants listed in exome variant server	0	0	3

## Discussion

Our study aimed at testing the current haploinsufficiency hypothesis for the autosomal dominant HSP type SPG8. As a first pertinent approach, we generated a murine *E430025E21Rik* allele that lacks an out-of-frame exon. As we found virtually no corresponding mRNA *in vivo*, probably because of nonsense-mediated decay [40], this alteration effectively generated a knockout allele. Contrary to what has been suggested [23], the heterozygous presence of such an inactivating mutation neither resulted in compensatory up-regulation of mRNA expression from the wild-type allele nor in substantially enhanced stability of the strumpellin protein. Heterozygous presence of a knockout allele therefore entails a reduction of the amount of strumpellin.

Near complete reduction of strumpellin levels by RNAi-mediated knockdown has been shown to severely destabilize other SHRC subunits [11, 12]. We show that the ~35 % reduction in strumpellin levels observed upon heterozygous knockout results in a similar degree of reduction for Fam21 levels. This is consistent with the proposed general dependence of SHRC subunit stability on proper assembly of the complex [11–13, 17].

Reduced abundance of SHRC in heterozygous strumpellin knockout cells likely results in correspondingly reduced overall SHRC activity. The physiological role of SHRC is not yet completely understood, but recent studies suggested an involvement in maintaining the structure/complexity of the endo- and lysosomal compartments [17], in inhibiting autophagy [19], but also in promoting autophagy [20, 21]. The effects of blocking SHRC function in these studies were rather severe and included collapsing of organelle compartments and aggregate formation. We did, however, not observe any evidence for abnormal endosomes, lysosomes, or autophagosomes/autolysosomes in heterozygous strumpellin knockout MAFs. We also found no increase in endosomal tubulation, i.e. a major consequence of (near) complete reduction of the major SHRC subunit *Wash1* [11, 23]. This suggests that, in contrast to complete SHRC inactivation, moderately reduced SHRC abundance/activity is enough to maintain grossly normal function of the corresponding organelle systems.

There were also no phenotypic consequences associated with a reduction of murine strumpellin/SHRC *in vivo*. One may argue that axon length and maximum age would render mice unsuited models for HSPs in the first place. Numerous recent studies, however, have shown impressive and clearly disease-relevant phenotypes in murine models for progressive axonopathies. These include knockouts of two genes that have been shown to cause pure dominant HSP by haploinsufficiency: for both *SPAST* (spastin protein, SPG4) and *REEP1* (REEP1 protein, SPG31) there is progressive gait impairment, the severity of which negatively correlates with the amount of wild-type protein available [26, 41, 42]. While reducing the amounts of either spastin or REEP1 is thus pathogenic, this does not seem to apply to strumpellin/SHRC.

Bona fide *KIAA0196* knockout mutations have thus far not been found in human HSP patients. There are, however, only few pertinent screens reported, and tools for detecting deleted alleles have been lacking. A second part of our study thus addressed the potential presence of inactivating large deletions. The application of a homemade MLPA assay to 240 HSP index patients failed to reveal this class of mutations. Again, the comparison to the haploinsufficiency HSP genes *SPAST* and *REEP1* is of interest, as both their mutation and variation spectra turn out to be very different from those of *KIAA0196* (Table 2). The genetic data available therefore further indicate that strumpellin knockout alleles are well tolerated when present heterozygously. The generalization of this, i.e. that heterozygous absence of any SHRC member is tolerated, is suggested by the fact that the DECIPHER database [http://decipher.sanger.ac.uk/] lists several heterozygous whole gene deletions for each SHRC member, but none of the corresponding cases is tagged with a clinical movement phenotype. Taken together, our *in vivo* and genetic data as well as currently available database entries do not support a relevance of haploinsufficiency for SPG8.

The major alternative to haploinsufficiency in dominantly inherited disease is a dominant negative effect of mutant on wild-type protein, thereby effectively leading to complete loss-of-function. However, the embryonic lethality of our homozygous knockout mice, which parallels early embryonic death of homozygous *Wash1* knockouts [17, 19], is not compatible with complete loss-of-function in otherwise healthy HSP patients. Against this background, we note a recent report of a recessive *KIAA0196* mutation affecting the very 3′ part of the coding sequence: the mutation, which is predicted to maintain only residual activity, does not confer an upper motoneuron phenotype, but results in cardiac, cerebellar, and craniofacial abnormalities [43]. These symptoms probably correspond to what is observed upon (incomplete) strumpellin knockdown in zebrafish [22].

Given that neither haploinsufficiency nor a dominant negative effect seem to mediate pathogenicity of HSP-associated strumpellin alterations, what does? We propose to consider a toxic gain-of-function effect caused by the accumulation of misfolded protein. Along this line, the interaction of strumpellin with the aggregation-prone VCP protein, and its presence in several neurodegeneration-associated intracellular deposits is noteworthy [22]. The fact that no aggregation has been observed upon overexpression of mutant strumpellin [23] may simply reflect the large differences in the time scale (several hours in cell culture vs. early adulthood clinical consequences in patients). Fully resolving the mutational mechanism in SPG8 will probably require appropriate knockin *in vivo* models.

## Conclusion

Our study does not lend support to the current haploinsufficiency hypothesis for SPG8, and also corroborates non-relevance of a dominant negative effect of mutant on wild-type strumpellin. It can thus be expected to initiate novel experimental approaches towards understanding and, eventually, treating SPG8. More generally, it emphasizes that determination of the mutational mechanism should be regarded an indispensable step for each genetically determined disorder.

## Additional files

Additional file 1: Figure S1.
*KIAA0196*-specific MLPA probes. Shown are exons against which probes were designed, plus neighbouring intronic sequence. The MLPA probe binding sequence is underlined. White underlined space indicates the ligation site. Marked in yellow is the stop codon in the terminal exon 29, while the nucleotide marked in red represents SNP rs188863489 which served as a positive control. Stretches of Ns indicate intronic sequence masked out by repeat masker [http://www.repeatmasker.org/]. (DOC 28 kb) Additional file 2: Figure S2. Visualization of lysosomes (LAMP-1), early endosomes (EEA1), and autophagosomes/lysosomes (LC3-RFP) by immunofluorescence (LAMP-1, EEA1) and transfection of an autofluorescent marker (LC3-RFP) in primary adult mouse fibroblast cultures. Scale bars: 20 μm. (TIFF 1055 kb)

## References

[1] Fink JK (2013). Hereditary spastic paraplegia: clinico-pathologic features and emerging molecular mechanisms. Acta Neuropathol.

[2] Schule R, Schols L (2011). Genetics of hereditary spastic paraplegias. Semin Neurol.

[3] Novarino G, Fenstermaker AG, Zaki MS, Hofree M, Silhavy JL, Heiberg AD (2014). Exome sequencing links corticospinal motor neuron disease to common neurodegenerative disorders. Science.

[4] Hedera P, Rainier S, Alvarado D, Zhao X, Williamson J, Otterud B (1999). Novel locus for autosomal dominant hereditary spastic paraplegia, on chromosome 8q. Am J Hum Genet.

[5] Valdmanis PN, Meijer IA, Reynolds A, Lei A, MacLeod P, Schlesinger D (2007). Mutations in the KIAA0196 gene at the SPG8 locus cause hereditary spastic paraplegia. Am J Hum Genet.

[6] de Bot ST, Vermeer S, Buijsman W, Heister A, Voorendt M, Verrips A (2013). Pure adult-onset spastic paraplegia caused by a novel mutation in the KIAA0196 (SPG8) gene. J Neurol.

[7] Bettencourt C, Morris HR, Singleton AB, Hardy J, Houlden H (2013). Exome sequencing expands the mutational spectrum of SPG8 in a family with spasticity responsive to L-DOPA treatment. J Neurol.

[8] Ishiura H, Takahashi Y, Hayashi T, Saito K, Furuya H, Watanabe M (2014). Molecular epidemiology and clinical spectrum of hereditary spastic paraplegia in the Japanese population based on comprehensive mutational analyses. J Hum Genet.

[9] Wang X, Yang Y, Wang X, Li C, Jia J (2014). A novel KIAA0196 (SPG8) mutation in a Chinese family with spastic paraplegia. Chin Med J (Engl).

[10] Jahic A, Kreuz F, Zacher P, Fiedler J, Bier A, Reif S (2014). A novel strumpellin mutation and potential pitfalls in the molecular diagnosis of hereditary spastic paraplegia type SPG8. J Neurol Sci.

[11] Derivery E, Sousa C, Gautier JJ, Lombard B, Loew D, Gautreau A (2009). The Arp2/3 activator WASH controls the fission of endosomes through a large multiprotein complex. Dev Cell.

[12] Jia D, Gomez TS, Metlagel Z, Umetani J, Otwinowski Z, Rosen MK (2010). WASH and WAVE actin regulators of the Wiskott-Aldrich syndrome protein (WASP) family are controlled by analogous structurally related complexes. Proc Natl Acad Sci U S A.

[13] Gomez TS, Billadeau DD (2009). A FAM21-containing WASH complex regulates retromer-dependent sorting. Dev Cell.

[14] Harbour ME, Breusegem SY, Antrobus R, Freeman C, Reid E, Seaman MN (2010). The cargo-selective retromer complex is a recruiting hub for protein complexes that regulate endosomal tubule dynamics. J Cell Sci.

[15] Seaman MN (2012). The retromer complex - endosomal protein recycling and beyond. J Cell Sci.

[16] Seaman MN, Gautreau A, Billadeau DD (2013). Retromer-mediated endosomal protein sorting: all WASHed up!. Trends Cell Biol.

[17] Gomez TS, Gorman JA, de Narvajas AA, Koenig AO, Billadeau DD (2012). Trafficking defects in WASH-knockout fibroblasts originate from collapsed endosomal and lysosomal networks. Mol Biol Cell.

[18] Piotrowski JT, Gomez TS, Schoon RA, Mangalam AK, Billadeau DD (2013). WASH knockout T cells demonstrate defective receptor trafficking, proliferation, and effector function. Mol Cell Biol.

[19] Xia P, Wang S, Du Y, Zhao Z, Shi L, Sun L (2013). WASH inhibits autophagy through suppression of Beclin 1 ubiquitination. EMBO J.

[20] King JS, Gueho A, Hagedorn M, Gopaldass N, Leuba F, Soldati T (2013). WASH is required for lysosomal recycling and efficient autophagic and phagocytic digestion. Mol Biol Cell.

[21] Zavodszky E, Seaman MN, Moreau K, Jimenez-Sanchez M, Breusegem SY, Harbour ME (2014). Mutation in VPS35 associated with Parkinson’s disease impairs WASH complex association and inhibits autophagy. Nat Commun.

[22] Clemen CS, Tangavelou K, Strucksberg KH, Just S, Gaertner L, Regus-Leidig H (2010). Strumpellin is a novel valosin-containing protein binding partner linking hereditary spastic paraplegia to protein aggregation diseases. Brain.

[23] Freeman C, Seaman MN, Reid E (2013). The hereditary spastic paraplegia protein strumpellin: characterisation in neurons and of the effect of disease mutations on WASH complex assembly and function. Biochim Biophys Acta.

[24] Pfaffl MW (2001). A new mathematical model for relative quantification in real-time RT-PCR. Nucleic Acids Res.

[25] Harbour ME, Breusegem SY, Seaman MN (2012). Recruitment of the endosomal WASH complex is mediated by the extended ‘tail’ of Fam21 binding to the retromer protein Vps35. Biochem J.

[26] Beetz C, Koch N, Khundadze M, Zimmer G, Nietzsche S, Hertel N (2013). A spastic paraplegia mouse model reveals REEP1-dependent ER shaping. J Clin Invest.

[27] Khundadze M, Kollmann K, Koch N, Biskup C, Nietzsche S, Zimmer G (2013). A hereditary spastic paraplegia mouse model supports a role of ZFYVE26/SPASTIZIN for the endolysosomal system. PLoS Genet.

[28] Irintchev A, Simova O, Eberhardt KA, Morellini F, Schachner M (2005). Impacts of lesion severity and tyrosine kinase receptor B deficiency on functional outcome of femoral nerve injury assessed by a novel single-frame motion analysis in mice. Eur J Neurosci.

[29] Sinning A, Liebmann L, Kougioumtzes A, Westermann M, Bruehl C, Hubner CA (2011). Synaptic glutamate release is modulated by the Na + −driven Cl-/HCO(3)(−) exchanger Slc4a8. J Neurosci.

[30] Seluanov A, Vaidya A, Gorbunova V (2010). Establishing primary adult fibroblast cultures from rodents. J Vis Exp.

[31] Schule R, Brandt E, Karle KN, Tsaousidou M, Klebe S, Klimpe S (2009). Analysis of CYP7B1 in non-consanguineous cases of hereditary spastic paraplegia. Neurogenetics.

[32] Bauer P, Stevanin G, Beetz C, Synofzik M, Schmitz-Hubsch T, Wullner U (2010). Spinocerebellar ataxia type 11 (SCA11) is an uncommon cause of dominant ataxia among French and German kindreds. J Neurol Neurosurg Psychiatry.

[33] Beetz C, Nygren AO, Schickel J, Auer-Grumbach M, Burk K, Heide G (2006). High frequency of partial SPAST deletions in autosomal dominant hereditary spastic paraplegia. Neurology.

[34] Ferreirinha F, Quattrini A, Pirozzi M, Valsecchi V, Dina G, Broccoli V (2004). Axonal degeneration in paraplegin-deficient mice is associated with abnormal mitochondria and impairment of axonal transport. J Clin Invest.

[35] Soderblom C, Stadler J, Jupille H, Blackstone C, Shupliakov O, Hanna MC (2010). Targeted disruption of the Mast syndrome gene SPG21 in mice impairs hind limb function and alters axon branching in cultured cortical neurons. Neurogenetics.

[36] Renvoise B, Stadler J, Singh R, Bakowska JC, Blackstone C (2012). Spg20−/− mice reveal multimodal functions for Troyer syndrome protein spartin in lipid droplet maintenance, cytokinesis and BMP signaling. Hum Mol Genet.

[37] Hazan J, Fonknechten N, Mavel D, Paternotte C, Samson D, Artiguenave F (1999). Spastin, a new AAA protein, is altered in the most frequent form of autosomal dominant spastic paraplegia. Nat Genet.

[38] Zuchner S, Wang G, Tran-Viet KN, Nance MA, Gaskell PC, Vance JM (2006). Mutations in the novel mitochondrial protein REEP1 cause hereditary spastic paraplegia type 31. Am J Hum Genet.

[39] Depienne C, Fedirko E, Forlani S, Cazeneuve C, Ribai P, Feki I (2007). Exon deletions of SPG4 are a frequent cause of hereditary spastic paraplegia. J Med Genet.

[40] Baker KE, Parker R (2004). Nonsense-mediated mRNA decay: terminating erroneous gene expression. Curr Opin Cell Biol.

[41] Tarrade A, Fassier C, Courageot S, Charvin D, Vitte J, Peris L (2006). A mutation of spastin is responsible for swellings and impairment of transport in a region of axon characterized by changes in microtubule composition. Hum Mol Genet.

[42] Kasher PR, De Vos KJ, Wharton SB, Manser C, Bennett EJ, Bingley M (2009). Direct evidence for axonal transport defects in a novel mouse model of mutant spastin-induced hereditary spastic paraplegia (HSP) and human HSP patients. J Neurochem.

[43] Elliott AM, Simard LR, Coghlan G, Chudley AE, Chodirker BN, Greenberg CR (2013). A novel mutation in KIAA0196: identification of a gene involved in Ritscher-Schinzel/3C syndrome in a First Nations cohort. J Med Genet.

[44] Goizet C, Depienne C, Benard G, Boukhris A, Mundwiller E, Sole G (2011). REEP1 mutations in SPG31: frequency, mutational spectrum, and potential association with mitochondrial morpho-functional dysfunction. Hum Mutat.

[45] Yip AG, Durr A, Marchuk DA, Ashley-Koch A, Hentati A, Rubinsztein DC (2003). Meta-analysis of age at onset in spastin-associated hereditary spastic paraplegia provides no evidence for a correlation with mutational class. J Med Genet.

[46] Beetz C, Schule R, Deconinck T, Tran-Viet KN, Zhu H, Kremer BP (2008). REEP1 mutation spectrum and genotype/phenotype correlation in hereditary spastic paraplegia type 31. Brain.

