# Supplementary material for: The spectrum of KIAA0196 variants, and characterization of a murine knockout: implications for the mutational mechanism in hereditary spastic paraplegia type SPG8
Source: Orphanet J Rare Dis. 2015 Nov 16;10:147. doi: 10.1186/s13023-015-0359-x (PMC4647479; doi:10.1186/s13023-015-0359-x)
Supplement: Additional file 1: Figure S1. — KIAA0196-specific MLPA probes. Shown are exons against which probes were designed, plus neighbouring intronic sequence. The MLPA probe binding sequence is underlined. White underlined space indicates the ligation site. Marked in yellow is the stop codon in the terminal exon 29, while the nucleotide marked in red represents SNP rs188863489 which served as a positive control. Stretches of Ns indicate intronic sequence masked out by repeat masker [http://www.repeatmasker.org/]. (DOC 28 kb) [file 13023_2015_359_MOESM1_ESM.doc]

## Supplementary File 1. *KIAA0196*-specific MLPA probes. Shown are exons against which probes were designed, plus neighbouring intronic sequence. The MLPA probe binding sequence is underlined. White underlined space indicates the ligation site. Marked in yellow is the stop codon in the terminal exon 29, while the nucleotide marked in red represents SNP rs188863489 which served as a positive control. Stretches of Ns indicate intronic sequence masked out by repeat masker [http://www.repeatmasker.org/].

Exon 06

TTTGAATCTTGAGGATATATTGAGGATATAGATGGACCTGTGATCATATCTGAAACTATATTGAGTCAAAGTAGAATGGAATTCTACTTTATTGGACTTTTCAGTTTCTAATATGTGTGTTTTGGGTCTTTGTTTCCCATTGTGAATATGACTCAGTGCTGCTCGATCTTCTGCTGATTCAAATATGGACGATATTTGTAAGCTGCTTCGAAGTACAGGTTATTCTAGCCAACCAGGTGCCAAAAGACCATCCAACTATCCCGAGAGCTATTTCCAGAGAGTGCCTATCA_ACGAATCCTTCATCAGTATGGTCATTGGTCGACTGAGATCTGATGATATTTACAACCAGGTGGGAATTAAATCTATTAAGACCTATTAAGACAGTTTAACAGACTGATTTCACTTCCCTCTTTAATTACTCCTTTCCTTCCTTCTTTCCCAAAAGAGTAAAAGCAGGTAGAACCTTTTAAAACCTTTTGAGCTACACCCCAGGATGATAAAGGAAAGAGGGTTATTAATTTTGCCAGAATCATAT

Exon 10

NNNNNNNNNNNNNNNNNNNNNNNNNNNNACTTTTTAAATGGTTAAAATGAAGGAAAATATTTAATAATTTATCTTATTTCATAGATATTTTCTACACATTTAGTAAGCAGGTAACACTTTTCCTGTTTCTAGCCTGTGACCCAAACAACAA_ACGCCTTCGTCAAATCAAGGACCAGATTCTAACAGACTCTCGGTACAATCCCAGGATCCTCTTCCAGCTGCTGTTAGATACTGCACAATTTGAGTTTATACTCAAAGAGGTAAAAATATTTGAATGAATATCACCACAGTCTTAGCTTTTTTATGCTTAAAAATATTTTTTAAAAAAAAGACTAGGATTGCTTGCTCTGTCTCTGATTCAAGGTACAGATTTGAGCTGTTGAGATAGTTCTTATAGTTAAAGGACAGGNNNNNNNNNNNNNNNNNNN

Exon 16

ACAGCTCTTTTACATTTTTCAGCATCCCCCTATATAGACAAATTAGGCATCCTAATATTTGTCAGCCAAGAAAAGTAAAACAGATGTTCATTGCGTTTCCTGCTTTCCTTTTCACTGAAGGTTTTGCAGA_TCATCCCAGAAAGCATGTTTACATCTCTTCTAAAGATCATAAAGCTTCAGACCCACGACATTATTGAAGTGCCTACCCGCCTGGACAAAGACAAGCTGAGGGACTATGCTCAGCTAGGCCCACGATACGAGGTGCTGTGTCCCTCTGACTTCTGCCTTTTCATATTAAAAACTAACAGTCATGAAATATCACAGGCCTGTGTCACCTGCCATATTCTGGGGACCACCAGACCAATTCTGACTTGTTGTCTCACATTTGCAATGGTAAACGCGTTTTATGATTTTAGTACAATCACACC

Exon 21

ATTGGGTGCCTGAATACAGAGGTTAGAAAACACAGATCATGTAACTCCTGTTCTTCATATTTGGTGCCGCATGTCCTGTTGAGTCCTAACATCTCTTTAAGGATGAATTCTGAGCCATTCTGGGGTTTTTGCATTGTTTTCATTTTAGAATGACATGTCACATAGACCAGCTGAACACTTGGTATGATATGAAAACTCATCAGGAAGTGACCAGCAGCCGCCTCTTCTCAGA_AATCCAGACCACCTTGGGAACCTTTGGTCTAAATGGCTTAGACAGGCTTCTGTGCTTTATGATTGTAAAAGAGTTACAGGTGAGCCTTTTGACTTTCATGCCTGTTTTCTGCCACCCTGGGGGAGAGGCAAGAACATATTTTAGTGGGCAGTTTGAACTCACTTAACCTTTCTTCCATGATTTCTTACACTGACTT

Exon 25

NNNNNNNNNNNNNNNNNNNNNNNNNNNNNNNNNNNNNNNNNNNTATATTAACTCTTGTATAAATTCATTTACTCACTAATGTTTATCTCCAGGGTTTTTCTTCCTTTAGGGCTCTCCTAGCAGACATTGAAGCCCACTATCAGGACCCTTCACTTCCTTACCCCA_AAGAAGATAACACACTTTTATATGAAATCACAGCCTATCTGGAGGCAGCTGGCATTCACAACCCACTGAATAAGGTGAGTAATTAAAATATATAATGGCGGGGATGGGGGTACAGAATGGTATTTGGCCCAGGTAACAGATTACTGTGCAGAAATACCAGAGGAACCTGGGTTCAGACCCCAGAGCCTTCATTCCATNNNNNNNNNNNNNNNNNNNNNNNNNNNNNNNNNNNNNNNNNNNNNNNNNNNNNNNNNNNNNNNNNN

Exon 29

AATTTTGTAATTGTTCAACTATAACACCATGAATAATTTAATTCTTTAATTTTTCAGGTTGCTGAAGCACATGTGCCTAATTTCATTTTTGATGAGTTCAGAACAGTGCTGTAACTGTTTTTCCTACTTCTTCAATGGAAGGATTGTCCTTAGATCTTCCCACCATCACAAATGAATTTGAAGATGAAAAGAA_ACTCAGTTGCTCATACAACTGCATTTTTTCTGTCTATTATGGGAAACATCAGACGTTCTGAGTAAGATATATCTCATGGCATTAGTTAATATAACTGATATTGTTTAAATCATGGTATTACATGCAATTTATATCAGATAAAAGCAGAACACATTTTTGTACTGCCTCTCTTAAATGCTGAATGTAACTGTTATGTATAAATCCATTTAGTTTTATGTTCTAAAGAACTATTTGTGCAACTCCAGATTTTCAGTAAAATAGTATTACTAGTACCCAA

Oligonucleotides representing left and right MLPA halfprobes as finally ordered (“…_right” with 5’ phosphorylation):

SPG8_Ex06_left: GGGTTCCCTAAGGGTTGGACCAACTATCCCGAGAGCTATTTCCAGAGAGTGCCTATCA

SPG8_Ex10_left: GGGTTCCCTAAGGGTTGGACCTGTTTCTAGCCTGTGACCCAAACAACAA

SPG8_Ex16_left: GGGTTCCCTAAGGGTTGGACCTGCTTTCCTTTTCACTGAAGGTTTTGCAGA

SPG8_Ex21_left: GGGTTCCCTAAGGGTTGGACCAGCAGCCGCCTCTTCTCAGA

SPG8_Ex25_left: GGGTTCCCTAAGGGTTGGACCTAGCAGACATTGAAGCCCACTATCAGGACCCTTCACTTCCTTACCCCA

SPG8_Ex29_left: GGGTTCCCTAAGGGTTGGACCACCATCACAAATGAATTTGAAGATGAAAAGAA

SPG8_Ex06_right: ACGAATCCTTCATCAGTATGGTCATTGGTCTAGATTGGATCTTGCTGGCAC

SPG8_Ex10_right: ACGCCTTCGTCAAATCAAGGACCAGATTCTAACAGACTCTCGGTCTAGATTGGATCTTGCTGGCAC

SPG8_Ex16_right: TCATCCCAGAAAGCATGTTTACATCTCTTCTAAAGATCATAAAGCTTCAGACCCACGTCTAGATTGGATCTTGCTGGCAC

SPG8_Ex21_right: AATCCAGACCACCTTGGGAACCTTTGGTCTAGATTGGATCTTGCTGGCAC

SPG8_Ex25_right: AAGAAGATAACACACTTTTATATGAAATCACAGCCTATCTGGTCTAGATTGGATCTTGCTGGCAC

SPG8_Ex29_right: ACTCAGTTGCTCATACAACTGCATTTTTTCTGTCTATTATGGTCTAGATTGGATCTTGCTGGCAC
