# Supplementary figures and images for: The spectrum of KIAA0196 variants, and characterization of a murine knockout: implications for the mutational mechanism in hereditary spastic paraplegia type SPG8
Source: Orphanet J Rare Dis. 2015 Nov 16;10:147. doi: 10.1186/s13023-015-0359-x (PMC4647479; doi:10.1186/s13023-015-0359-x)

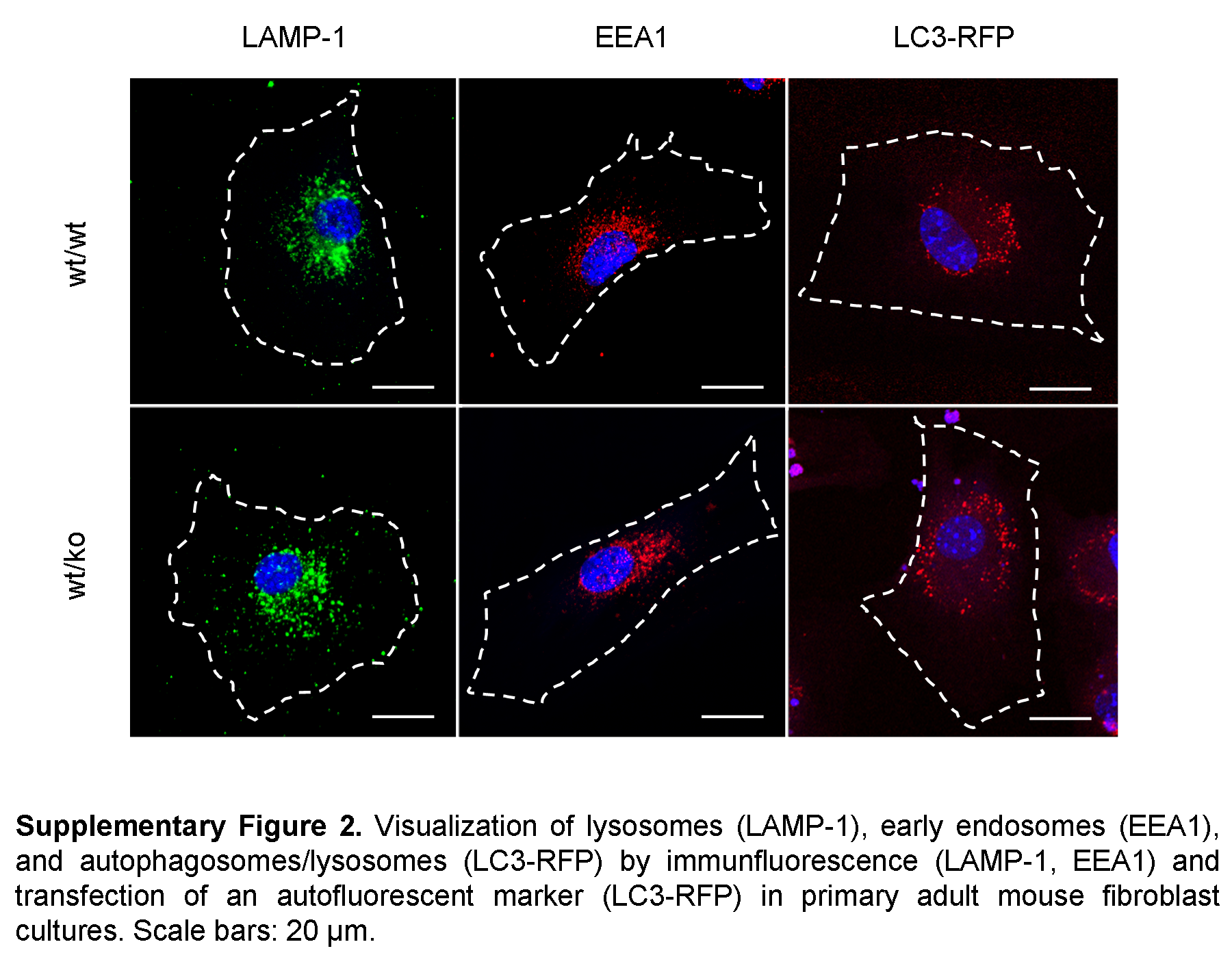

Supplement: Additional file 2: Figure S2. — Visualization of lysosomes (LAMP-1), early endosomes (EEA1), and autophagosomes/lysosomes (LC3-RFP) by immunofluorescence (LAMP-1, EEA1) and transfection of an autofluorescent marker (LC3-RFP) in primary adult mouse fibroblast cultures. Scale bars: 20 μm. (TIFF 1055 kb) [file 13023_2015_359_MOESM2_ESM.tif]
